# Structural basis of antigen recognition: crystal structure of duck egg lysozyme

**DOI:** 10.1107/S2059798317013730

**Published:** 2017-10-25

**Authors:** David Brent Langley, Ben Crossett, Peter Schofield, Jenny Jackson, Mahdi Zeraati, David Maltby, Mary Christie, Deborah Burnett, Robert Brink, Christopher Goodnow, Daniel Christ

**Affiliations:** a Immunology Division, Garvan Institute of Medical Research, 384 Victoria Street, Darlinghurst, NSW 2010, Australia; b The Mass Spectrometry Core Facility, The University of Sydney, The Hub, Charles Perkins Centre, Building D17, Camperdown, NSW 2006, Australia; c Molecular, Structural and Computational Division, Victor Chang Cardiac Research Institute, 405 Liverpool Street, Darlinghurst, NSW 2010, Australia; d St Vincent’s Clinical School, University of New South Wales, Darlinghurst, NSW 2010, Australia

**Keywords:** duck egg lysozyme, hen egg lysozyme, glycosyl hydrolase, antigen recognition

## Abstract

Duck egg lysozyme (DEL) is a widely used model antigen owing to its capacity to bind with differential affinity to anti-chicken egg lysozyme antibodies. However, no structures of DEL have so far been reported, and the situation had been complicated by the presence of multiple isoforms and conflicting reports of primary sequence. Here, the structures of two DEL isoforms from the eggs of the commonly used Pekin duck (*Anas platyrhynchos*) are reported. Using structural analyses in combination with mass spectrometry, non-ambiguous DEL primary sequences are reported. Furthermore, the structures and sequences determined here enable rationalization of the binding affinity of DEL for well documented landmark anti-lysozyme antibodies.

## Abstract 2

Lysozyme isoforms purified from duck eggs have been characterized by crystallography, mass spectrometry and their capacity to bind landmark antibodies.

## 1. Introduction   

Lysozymes purified from bird eggs are one of the best bio­logically, immunologically and structurally characterized families of proteins. The Protein Data Bank (PDB) currently contains approximately 650 entries containing chicken-type (C-type) hen egg-white lysozyme (HEL), HEL variants or complexes involving the HEL entity, in some cases at extremely high resolution. A far more limited number of PDB entries contain C-type lysozymes derived from a diverse range of other species, including turkey (Sarma & Bott, 1977 ▸), trout (Karlsen *et al.*, 1995 ▸), human (Artymiuk & Blake, 1981 ▸) and even echidna (Guss *et al.*, 1997 ▸). The C-type lysozyme is distinct at the sequence, structural and immunological levels from two other classes of lysozyme: the form purified from goose eggs (‘G-type’; Canfield & McMurry, 1967 ▸; Prager *et al.*, 1974 ▸) and that encoded by bacteriophages (T4-type; Weaver & Matthews, 1987 ▸), neither of which will be discussed at length in this report. Surprisingly, despite the enzyme purified from duck eggs being extensively studied for its biochemical and antibody-recognition properties, until now no PDB entries have existed for C-type lysozyme derived from ducks.

The fact that multiple duck egg isoforms could be separated using ion-exchange resins was established in the mid 1960s by, amongst others (Imanishi *et al.*, 1966 ▸), the Parisian duo of Jacqueline and Pierre Jollès (Jollès *et al.*, 1965 ▸), who had just previously helped to establish the amino-acid sequence of the chicken enzyme (Jollès *et al.*, 1963 ▸, 1964 ▸), the landmark structure of which was solved shortly afterwards in 1965 (Blake *et al.*, 1965 ▸). They noted that one of the key differences between the chicken and duck enzymes was the progressive increase in the amount of arginine found in the duck isoforms (Jollès *et al.*, 1967 ▸), and they subsequently determined the entire primary sequences of two of the three isoforms, which they termed Duck II and Duck III (Hermann *et al.*, 1971 ▸, 1973 ▸). Whilst the Jollès group had been working with the ‘Khaki’ strain of the domestic duck (an English strain of the mallard, *Anas platyrhynchos*), in California, Prager and Wilson, working with the Pekin strain (the common Asian domestic strain of *A. platyrhynchos*), independently characterized the three isoforms (termed Duck A, B and C) and suggested there were likely to be three alleles for the gene, as no more than two isoforms were found in any given egg (Prager & Wilson, 1971 ▸). Furthermore, their amino-acid analysis suggested that the primary sequences might be slightly different between the Khaki and Pekin strains, but that otherwise Duck A, B and C were analogous to Duck I, II and III, respectively. The most comprehensive investigation of the primary sequence of the duck isoforms *via* amino-acid sequencing was published in 1982 by the Japanese group of Kondo, Fujio and Amano (Kondo *et al.*, 1982 ▸), who processed some 450 Pekin duck eggs and sequenced all three isoforms (which they termed DL-1, DL-2 and DL-3), with their results broadly agreeing with the amino-acid distributions published by Prager and Wilson. The genome for Pekin duck was sequenced in 2013 (Huang *et al.*, 2013 ▸); however, the sequence was derived entirely from a single individual, and only a single allele is presented for the gene in the database, which is essentially identical to the DL-2 primary amino-acid sequence of Kondo and coworkers, albeit with two asparagine/aspartic acid discrepancies. In general, the duck isoforms contain approximately 20 amino-acid substitutions (of 129 positions) relative to the chicken enzyme, and were thus considered to be likely to maintain essentially the same fold.

The structure of Khaki Duck II seems to have been very nearly solved in 1972, as reported by Berthou, Laurant and Jollès (Berthou *et al.*, 1972 ▸). Large crystals of the Duck II protein were grown, preliminary diffraction data were collected and the space group was determined; heavy-atom soaks were even performed and the occupancies of Hg atoms were refined. For reasons that are unknown, no structure, if indeed it were completed, has been made publically available.

Although no crystal structure of any of the duck lysozymes was available, variants obtained from ducks (species and isoform often unspecified) have nevertheless proved to be useful immunological tools as counterpoints to the chicken enzyme in rationalizing antigen–antibody interfaces and affinities (Smith-Gill *et al.*, 1982 ▸; Lavoie *et al.*, 1992 ▸) and, more recently, in studying aspects of the immune response such as immune tolerance (Shokat & Goodnow, 1995 ▸), complement activation (Manderson *et al.*, 2006 ▸), affinity maturation of B-cells in the germinal centre (Allen *et al.*, 2007 ▸; Phan *et al.*, 2009 ▸), apoptosis and B-cell differentiation into effector-cell classes (Taylor *et al.*, 2015 ▸), and the differentiation of T-follicular helper cells (Lee *et al.*, 2015 ▸). To aid such investigations, we have characterized the primary structures of lysozyme isoforms from Pekin duck by mass spectrometry and solved their three-dimensional structures.

## 2. Materials and methods   

### 2.1. Genealogy   

Duck eggs from Pekin ducks were procured from Talsar Egg and Produce Supplies, Sydney; genealogy is traceable to the importation of Grimaud commercial strains from France.

### 2.2. Purification   

The whites of a dozen eggs were separated from the yolks and pooled (approximately 400–500 ml, pH ∼9.5), diluted with water to a volume of 3 l (pH ∼9.0) and then passed three times through two sheets of muslin to remove chalazae and thick albumin (approximately 200 ml was discarded). The pH was adjusted to approximately 9.5 by the addition of *N*-cyclohexyl-2-aminoethanesulfonic acid (CHES) to 50 m*M*. Approximately 50 ml of carboxymethyl ion-exchange resin (CM-650M, Toyopearl) was then added and the slurry was mixed for 2 h before stationary incubation at room temperature (RT) for 30 min, allowing the beads to settle, before the bulk of the liquid and fine precipitate was decanted and discarded. The slurry was loaded into a 150 ml syringe plugged with cotton and washed with 0.5 l of 50 m*M* CHES pH 9.5; the lysozyme-enriched fraction was then step-eluted with the same buffer spiked with 500 m*M* NaCl. Following dialysis against 50 m*M* Tris pH 8.8, 50 m*M* NaCl, the sample was loaded onto a pre-poured CM column (∼8.5 × 2.5 cm) and eluted using a linear 50–450 m*M* NaCl gradient (∼800 ml, collecting ∼100 × 6 ml fractions). Three distinct lysozyme-containing protein peaks were eluted (which we term DEL-I, DEL-II and DEL-III). Pooled fractions from each peak were purified further *via* gel filtration (S200 26/60 column, GE Healthcare) eluted with buffer consisting of 25 m*M* Tris pH 8.0, 150 m*M* NaCl. Samples were concentrated to approximately 10 mg ml^−1^ for crystallization trials.

### 2.3. Crystallization   

Initial crystallization conditions were obtained using commercial sparse-matrix screens (JCSG-*plus* and PACT *premier* from Molecular Dimensions, and SaltRx HT from Hampton Research), whereby 400 nl protein solution and an equal volume of well solution were combined in 96-well sitting-drop plates using a Mosquito liquid-handling robot (TTP Labtech). Optimization of conditions was performed in 24-well plates in hanging-drop format, where 2 µl well solution was combined with 2 µl protein solution. Crystals typically grew over several weeks at 20°C. DEL-I crystals with plate morphology were grown using a well solution consisting of 200 m*M* KSCN, 100 m*M* bis-tris propane pH 8.5, 20% PEG 3350 (essentially PACT *premier* condition H4). Two forms of DEL-III crystals were obtained: one (cubic morphology; DEL-IIIc) employing well conditions consisting of 100 m*M* NH_4_H_2_PO_4_, 100 m*M* Tris pH 8.8, 45% 2-methyl-2,4-pentanediol (based on JCSG-*plus* condition A11) and the other (plate morphology; DEL-IIIo) employing conditions consisting of 2.4 *M* (NH_4_)_2_HPO_4_, 100 m*M* Tris pH 8.5 (based on SaltRx condition E4). Crystals were flash-cooled by plunging them into liquid nitrogen.

### 2.4. Diffraction data, structure solution and refinement   

Diffraction data were collected at 100 K on beamline MX2 at the Australian Synchrotron. The diffraction data were indexed and integrated using *iMosflm* (Battye *et al.*, 2011 ▸), the space group was determined with *POINTLESS* (Evans, 2011 ▸) and scaling was performed with *AIMLESS* (Evans & Murshudov, 2013 ▸). Structures were solved *via* molecular replacement using *Phaser* (McCoy *et al.*, 2007 ▸) employing HEL as the search model. Rigid-body and restrained *B*-factor refinement were performed with *REFMAC*5 (Murshudov *et al.*, 2011 ▸), which is part of the *CCP*4 suite of crystallographic software. In the case of DEL-I and the orthorhombic form of DEL-III, the resolution was sufficient to justify the use of anisotropic *B* factors. Models were inspected and compared with electron-density maps, and where necessary modified, using *Coot* (Emsley *et al.*, 2010 ▸). Validation was performed using the *MolProbity* server (Chen *et al.*, 2010 ▸). Data-collection and refinement details are shown in Table 1 ▸.

### 2.5. Mass spectrometry   

To obtain the mass of the intact protein, solutions of the purified protein were diluted in 10%(*v*/*v*) acetonitrile/0.1%(*v*/*v*) formic acid to a concentration of 0.5 mg ml^−1^ and infused into a Orbitrap Fusion mass spectrometer (Thermo Fisher Scientific). The acquired mass spectrum was deconvoluted using *QualBrowser* (Thermo Fisher Scientific). To obtain more detailed sequence information, selected bands were excised from an SDS–PAGE gel and destained in a 60:40 solution of 40 m*M* ammonium bicarbonate pH 7.8:100% acetonitrile for 1 h. Samples were reduced with 40 m*M* dithiothreitol for 30 min. The buffer was then exchanged and the samples were alkylated with 80 m*M* iodoacetamide for 30 min. Gel pieces were vacuum-dried and then rehydrated with either 12 ng µl^−1^ porcine trypsin or 12 ng µl^−1^ of the serine protease isolated from *Lysobacter enzymogenes* (Lys-C) (both from Promega) at 4°C for 1 h. Excess trypsin/Lys-C was removed and 10 µl of 40 m*M* ammonium bicarbonate was added prior to incubation overnight at 37°C. A small portion of the peptide solution was then mixed with α-hydroxycinnamic acid and spotted onto a MALDI target plate. A mass spectrum was then collected using a QSTAR Elite Q-TOF mass spectrometer (Sciex) and the observed peaks were manually compared with the predicted peptide sequences. The remainder of the peptides were concentrated and desalted using C18 Zip-Tips (Millipore) as per the manufacturer’s instructions. Peptides were eluted into a 96-well plate and vacuum-dried. Prior to analysis, the peptides were reconstituted in 20 µl 3%(*v*/*v*) acetonitrile, 0.1%(*v*/*v*) formic acid and briefly sonicated. Samples were separated by nano-LC using an Eksigent 415 UHPLC system (Sciex) coupled to an in-house-built fritless 75 µm × 20 cm column packed with ReproSil Pur 120 C18 stationary-phase resin (1.9 µm; Dr Maisch GmbH, Germany). The LC mobile-phase buffers were buffer *A* consisting of 0.1%(*v*/*v*) formic acid and buffer *B* consisting of 80%(*v*/*v*) acetonitrile/0.1%(*v*/*v*) formic acid. Peptides were eluted using a linear gradient over 9–30 min. Mass spectra were then acquired using a 6600 Q-TOF mass spectrometer (Sciex). Up to 20 of the most abundant ions were sequentially isolated and fragmented and a product ion scan was collected. Ions selected for LCMS were dynamically excluded for 20 s. Raw data were converted to a peak list using *MS Data Converter* (Sciex) and the peak list was used to search a custom database using *Mascot* v.2.4 (Matrix Science, London, England). The settings deemed permissible were trypsin or Lys-C, two missed cleavages per peptide, a mass tolerance of 25 p.p.m. and variable oxidation for methionine, deamidation for glutamine/asparagine and carbamidomethyl cysteine. The peak assignment in LCMS spectra with significant (*p* < 0.05) *Mascot* peptide scores were manually reviewed to confirm putative peptide sequences.

### 2.6. Fab expression and purification   

HyHEL5 and HyHEL10 heavy- and kappa light-chain Fab sequences were synthesized and cloned into the pCEP4 expression vector (Thermo Fisher Scientific) *via* KpnI and BamHI restriction sites. Heavy chains were C-terminally His-tagged for purification purposes. Fab fragments were transiently expressed using the Expi293 Expression System (Thermo Fisher Scientific) according to the manufacturer’s recommendations using a 1:2 heavy-chain to light-chain ratio. Fab fragments were purified from cell-culture supernatant using HisTrap FF Crude columns (GE Healthcare) according to the manufacturer’s instructions. After dialysis against phosphate-buffered saline (PBS), Fab fragments were concentrated using spin filters (EMD Millipore) and analysed by SDS–PAGE, and their concentrations were determined by spectroscopy (absorbance at 280 nm).

### 2.7. Affinity measurements   

Affinity measurements between antibody Fab fragments and chicken and duck lysozymes were determined using Bio-Layer Interferometry (BLItz, ForteBio), essentially as previously described (Sabouri *et al.*, 2014 ▸). Briefly, purified HyHEL5 and HyHEL10 Fabs in PBS were biotinylated with EZ-Link NHS-PEG4-Biotinylation reagent (Thermo Fisher Scientific) at a biotin:protein ratio of 5:1. Free biotin was removed by passage through a ZebaSpin gel-filtration column (Thermo Fisher Scientific) equilibrated in PBS. Streptavidin biosensors (ForteBio) were rehydrated in PBS containing 0.1%(*w*/*v*) BSA for 1 h at RT. Biotinylated Fab was loaded onto the sensors ‘online’ using an advanced kinetics protocol. Purified duck and chicken (Sigma) lysozymes were then passaged on and off the Fab-coated sensor at a range of concentrations, allowing global fits of the binding kinetics to be determined using the *BlitzPro* 1.2.1.3 software (ForteBio).

## 3. Results   

**Figure 1:**
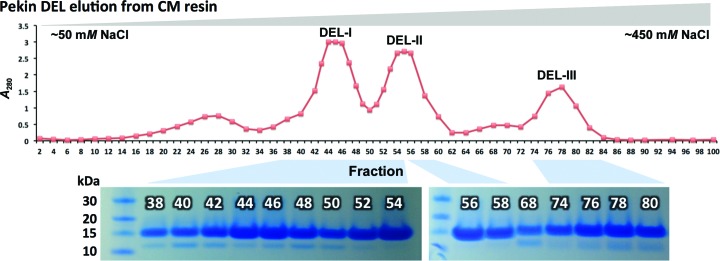
Elution profile of Pekin DEL fractions from CM ion-exchange resin (top) in response to a linear salt gradient (50–450 m*M* NaCl, left to right). Also shown are SDS–PAGE examinations of certain fractions (bottom).

**Figure 2:**
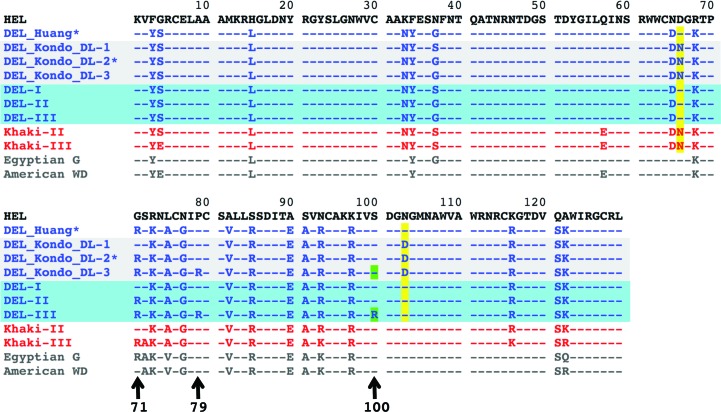
Alignment of the primary sequence of HEL (black text) with Pekin DEL sequences found in the literature (blue text; ‘DEL_Huang’ and ‘DEL_Kondo_’), with those structurally and/or confirmed by mass spectrometry described here (shaded blue; DEL-I, DEL-II ad DEL-III) as well as with sequences available for Khaki duck (red text), Egyptian goose and American wood duck (both grey text). Amino-acid identities are identical to HEL (indicated by a dash) unless explicitly stated. Ambiguities at positions 66 and 103 are highlighted in yellow, whilst arrows indicate the additional arginines in DEL sequences at positions 71, 79 and 100 (highlighted in green). Although derived by different means, the sequences of DEL_Huang and DEL_Kondo_DL-2 (marked with asterisks) effectively describe the same species (DEL-II).

The crystal structures of DEL isoforms I and III, purified from Pekin duck eggs and separated from each other using ion-exchange resin (Fig. 1 ▸), were solved (Tables 1 ▸ and 2 ▸). The resolutions of these structures were sufficient to unambiguously observe electron density for most amino acids, including their side chains. This allowed direct comparison with amino-acid sequences described in the literature which were obtained from a combination of amino-acid sequencing [as performed by Kondo *et al.* (1982 ▸), who describe all three isoforms], mass spectrometry [as performed by Takao *et al.* (1984 ▸), who also describe all three isoforms] and DNA sequencing [as performed by Huang *et al.* (2013 ▸), where just the one isoform is described]. Additional comparison could be made with the primary sequences of other duck species for which amino-acid sequences have been determined, including Egyptian goose (*Alopochen aegypticus*, actually a shelduck) and American wood duck (*Aix sponsa*) (UniProt entries P84496 and Q7LZQ2, respectively; Fig. 2 ▸).

An alignment of amino-acid sequences for HEL and Pekin DELs, both previously published and refined in this study, is shown in Fig. 2 ▸. At the time of writing, the nomenclature for the Pekin duck sequences as accessed *via* UniProt (Bateman *et al.*, 2015 ▸) is somewhat ambiguous. The DNA sequence-derived entry of Huang *et al.* (2013 ▸) (U3J0P1) is titled Lysozyme C-1, although the arginine content would imply that it is equivalent to the DL-2 sequence of Kondo *et al.* (1982 ▸) (entry P00705), which is also titled Lysozyme C-1. Hence, UniProt effectively contains two entries for the ‘middle’ of three DELs with respect to arginine content, but lacks entries for Pekin DEL-I and DEL-III. Although an entry titled Lysozyme C-3 does exist (entry P00706), this reports the Khaki Duck III sequence, as opposed to that from the Pekin strain. The DEL-II sequences are largely in agreement, with the main discrepancy being the identities of residues 66 and 103 (Fig. 2 ▸, yellow highlights), whereby an aspartic acid residue is swapped for an asparagine or *vice versa*. The mass-spectrometric analysis of Takao *et al.* (1984 ▸) supports the assignment of asparagine at position 103, but their data are more ambiguous with regards position 66. The electron-density maps for DEL-I and DEL-III, despite being at high resolution and with resolvable side chains at these positions, do not permit unequivocal discrimination of amino-acid identity, even in the context of hydrogen-bonding networks with surrounding water molecules.

**Figure 3:**
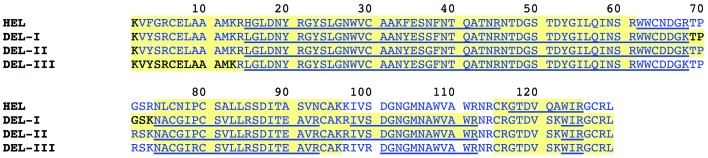
Summary of mass-spectrometric analyses to support the proposed sequences for DEL-I, DEL-II and DEL-III. Sequences in blue text match the predicted endopeptidase Lys-C peptide masses, while underlined sequences match the predicted tryptic peptide masses. Sequences highlighted in pale yellow were matched to an MSMS spectrum for that sequence.

To investigate these apparent sequence variations, as well as to shore up the identity of amino-acid positions for which clear side-chain density was lacking, different mass-spectrometric approaches were employed. One strategy was to digest each of the proteins with either trypsin or endoprotease Lys-C and compare the observed peptide mass spectra with theoretical peptide masses: so-called peptide mass fingerprinting. Fig. 3 ▸ shows sequences where observed peaks matched the theor­etical mass of Lys-C peptides in blue, while sequences where observed masses matched tryptic peptides are underlined. A variation of this approach was to take the same protease digests and analyse them by liquid chromatography-tandem mass spectrometry (LCMS). The shaded sequences in Fig. 3 ▸ indicate where tandem mass-spectrometric data matched the predicted sequence.

Overall, good coverage of the duck lysozymes was obtained, confirming that position 66 was likely to be aspartic acid (there is zero evidence for aspargine), as indicated by the DNA sequence of Huang *et al.* (2013 ▸), whilst position 103 showed a mixed population of asparagine and deamidated asparagine/aspartic acid, suggesting once again that the DNA-sequence data were likely to be correct for this position. Hence, positions 66 and 103 are identical not only to chicken but also Egyptian goose and American wood duck sequences (Fig. 2 ▸).

An additional whole-protein (or intact) mass analysis was also performed (Table 3 ▸), which returned masses that were in good agreement (<5 p.p.m.) with predictions derived from the sequences shown in Fig. 3 ▸. Although no high-quality crystals were obtained for DEL-II, mass spectrometry (peptide and whole protein) facilitated the primary-sequence assignment presented in Figs. 2 ▸ and 3 ▸.

**Figure 4:**
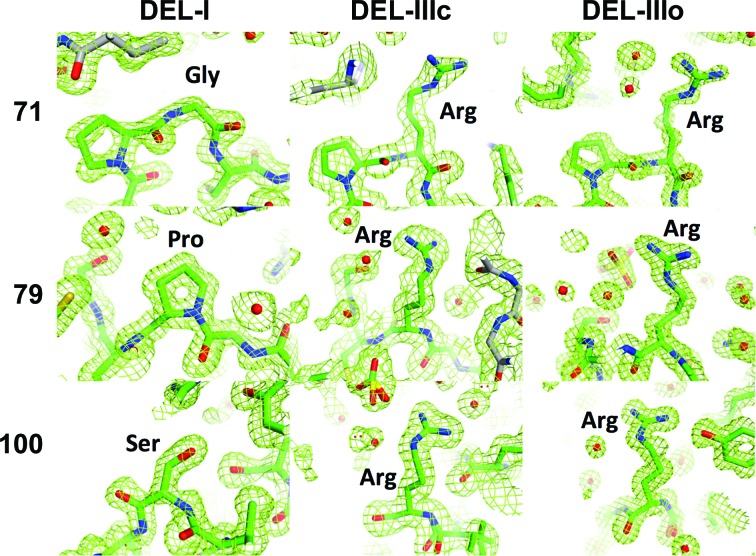
Composite OMIT 2*F*_o_ − *F*_c_ electron density (green mesh) contoured at 1σ for amino-acid positions 71, 79 and 100 for Pekin duck DEL-I and for cubic (DEL-IIIc) and orthorhombic (DEL-IIIo) crystal forms of DEL-III.

The most striking difference between the structures (and sequences) of DEL-I, DEL-II and DEL-III was the incremental number of arginine residues present in the proteins (Fig. 2 ▸, three of which are marked by arrows). DEL-I clearly contains a glycine at position 71 (Fig. 4 ▸, top left), as found in Kondo and coworkers’ DL-1 and in HEL. However, in DEL-III (and in Kondo and coworkers’ DL-3) residue 71 is clearly arginine (Fig. 4 ▸, top). Position 79 is a proline in Kondo and coworkers’ DL-I (and HEL) but is clearly an arginine in DEL-III (Fig. 4 ▸, middle row) and Kondo and coworkers’ DL-3 (but not in Kondo and coworkers’ DL-2, as confirmed by mass spectrometry). These differences, in and of themselves, would explain the sequential elution of DEL-I, DEL-II and DEL-III from CM resin when challenged with increasing salt. However, the electron density for DEL-III contained a surprise: position 100 was also very clearly an arginine residue as opposed to the serine observed in DEL-I and as previously described in DEL (and HEL) sequences (Fig. 4 ▸, bottom row). This additional unexpected arginine was present in both crystal forms of DEL-III, and was subsequently verified by mass spectrometry.

**Figure 5:**
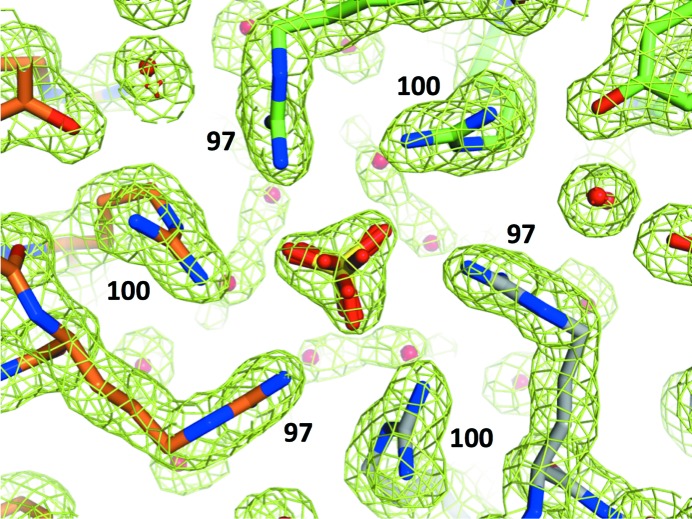
DEL-III cubic space group special position occupied by a phosphate anion which contacts Arg97 and Arg100. Composite OMIT 2*F*_o_ − *F*_c_ electron density (green mesh) contoured at 1σ as viewed down the crystallo­graphic threefold special position axis. Pairs of arginines projected from different lysozyme molecules are coloured green, grey and orange.

One of the crystal forms of DEL-III was also of interest as it crystallized in a cubic space group. The reason for this is related to the fact that position 100 comprises the additional arginine residue. Numerous phosphate anions, present in the crystallization condition, can be modelled, including several at crystallographic special positions (corner of the cube at the threefold symmetry axis), which act as crystal contacts that help to glue the crystal together. These phosphate positions are contacted by the guanidinium tips of three pairs of arginine side chains (residues 97 and 100); each pair is projected by a different lysozyme molecule within the crystal (Fig. 5 ▸).

To complement the newly available structures of DEL-I and DEL-III, and the primary sequence of DEL-II (essentially an S37G and G71R double mutant of DEL-I), we used bio-layer interferometry to assess the binding of these duck lysozymes to the Fab arms of the well characterized HEL-binding antibodies HyHEL5 and HyHEL10. All duck lysozymes displayed approximately a two orders-of-magnitude decrease in affinity for HyHEL5 relative to the chicken enzyme (Table 4 ▸). DEL-I and DEL-II also bound HyHEL10 with a similar affinity, which represented at least a three orders-of-magnitude decrease compared with HEL. In contrast, binding of HyHEL10 was essentially eliminated altogether in the case of DEL-III (Table 4 ▸).

## 4. Discussion   

We have solved the crystal structures of two of the three isoforms of lysozyme purified from Pekin duck eggs (DEL-I and DEL-III). The duck egg lysozyme structures have the basic fold of the C-type lysozyme characteristic of HEL. When lysozyme from duck eggs was first analyzed, it was noted that the most striking differences between the enzymes from ducks and chickens, apart from ducks having multiple isoforms, were the complete absence of any histidine residues in the duck variants (which at one stage was incorrectly suspected of being a requisite for catalysis) and the increased amounts of arginine relative to the chicken counterpart. These findings have been verified by our structures of DEL-I and DEL-III. Both contain a leucine residue in place of histidine at position 15, and whilst the enzyme from chicken contains 11 arginine residues, DEL-I contains 13, DEL-II contains 14 and DEL-III contains 16 (not 15 as expected). The single difference in charge between DEL-I and DEL-II, and an additional two positive charges between DEL-II and DEL-III, perhaps better explains the elution profile observed in response to increasing salt from CM ion-exchange resin; the peaks for DEL-I and DEL-II partially overlap, while clear separation exists between the peaks containing DEL-II and DEL-III (Fig. 1 ▸).

Residues 66 and 103 have consistently been problematic in their assignment, and not just in the case of duck lysozymes. Frequently, amino-acid sequences derived by Edman degradation have been revised either upon the publication of the DNA sequence or mass-spectrometric data. For example, Canfield’s original HEL sequence (Canfield, 1963 ▸) was later corrected, with Asp103 revised to Asn103 (Jung *et al.*, 1980 ▸). Indeed, in the sequence report of Kondo *et al.* (1982 ▸) the authors note that ‘the Asn-Gly (66–67) bond caused difficulty in sequence analysis’. In both cases we have modelled residues 66 and 103 reflecting the DNA sequence of Huang *et al.* (2013 ▸), consistent with our mass-spectrometric data (Asp66 and Asn103). It is now known that the frequency of non-enzymatic asparagine deamidation is particularly influenced by the adjacent C-terminal residue, with Asn-Gly being most susceptible (Wright, 1991 ▸), as is the case at positions 103–104. This causes additional complications in mass-spectrometric analysis. For example, during LCMS analysis around 50% of the matched spectra indicated that Asn103 was deamidated, which could be interpreted as Asp103. We concluded that Asn103 is the correct assignment and that the high level of deamidation is an experimental artifact. Conversely, in position 66 we observed no spectra matching an Asn66 assignment but many examples consistent with Asp66. These data are also consistent with the intact protein analysis (Table 3 ▸).

The additional arginine residue clearly identified at position 100 in our structure of DEL-III (Fig. 4 ▸) was not anticipated by prior sequence information, nor by mass spectrometry; Takao *et al.* (1984 ▸) don’t report coverage of DEL-III at this position. This discrepancy might reflect a variation in Pekin strains compared with those used by Kondo *et al.* (1982 ▸) in Japan. However, their publication contains some evidence that the duck strains might not, in fact, be different in this regard; they note that ‘non-specific cleavage with trypsin was observed at the Ser-Asp (100–101) bond’, suggesting that a positively charged (albeit poorly cleaving or somewhat inaccessible) residue might have indeed been at position 100.

**Figure 6:**
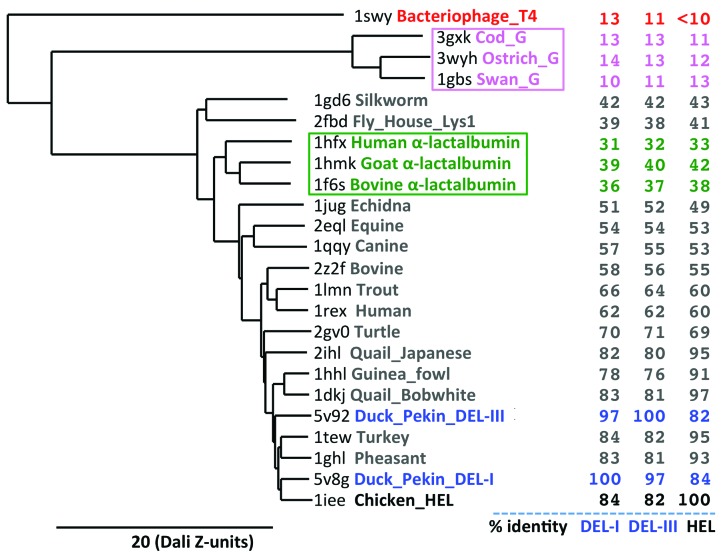
Structural fold dendogram of 24 structures encompassing the lysozyme family output by the *DALI* structural homology server (Holm & Laakso, 2016 ▸) and displayed using *TreeDyn* (Chevenet *et al.*, 2006 ▸). The *x* axis pertains to structural similarity in units of *DALI* *Z*-score. Percentage amino-acid identities relative to DEL-I, DEL-III and HEL are shown in the right-hand columns, respectively.

The structures of Pekin DEL-I and DEL-III can be compared not only with HEL, but also with the (surprisingly small) ensemble of lysozymes found in other organisms for which structures have also been determined. A protein-fold ‘all-on-all’ dendogram (Fig. 6 ▸), constructed using the *DALI* protein structure-comparison server (Holm & Laakso, 2016 ▸), reveals both structures to be closely related within a clade containing lysozymes from guinea fowl through chicken, despite the amino-acid identity for DEL-I and DEL-III relative to other members being considerably lower (∼80–85%) than for other species within the clade (all >90% with respect to each other) (Fig. 6 ▸, right-hand column). Outside of this clade amino-acid identities decrease to the ∼50–60% bracket for mammalian representatives and then decrease again upon comparison with lysozyme-like moieties such as the evolutionarily related mammalian α-lactalbumins (∼30–40%, green box and text), which maintain the same basic fold and arrangement of disulfide-bond pairs but lack the same enzymatic bacteriolytic activity. Insect-gut lysozymes are structurally more distantly related again (∼40% identity), but the C-type lysozymes (Fig. 6 ▸, silkworm through chicken) are vastly different in fold to the G-type or T4-type lysozymes (Fig. 6 ▸, top, pink and red, respectively; included for completeness).

**Figure 7:**
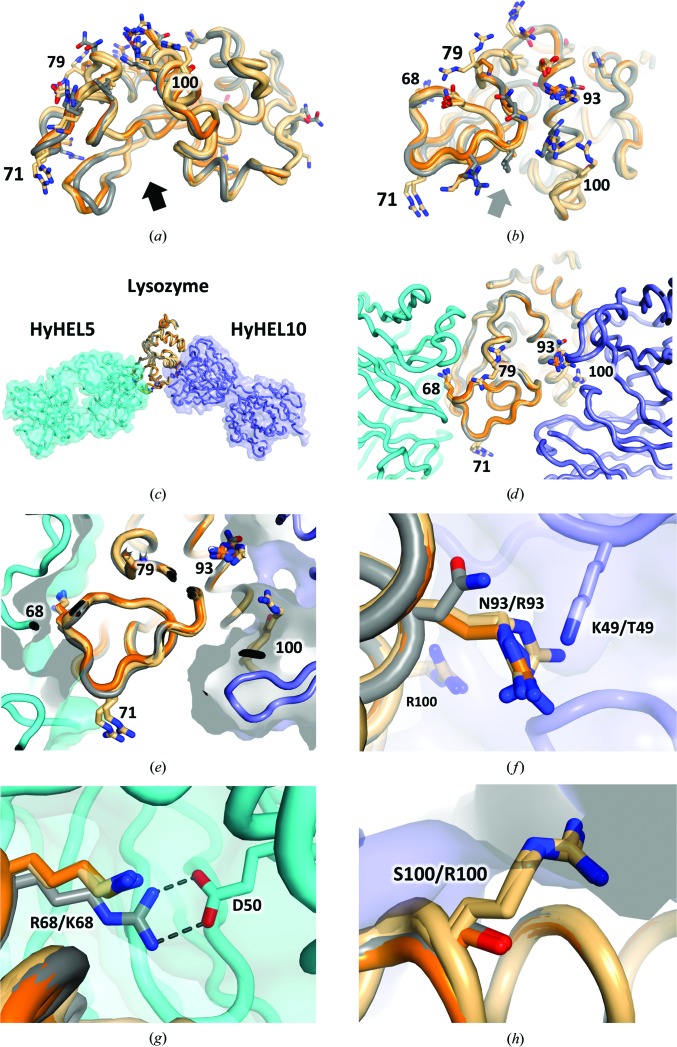
Duck lysozymes superposed on the chicken enzyme. (*a*, *b*) Cartoons of Pekin DEL-I (orange) and two crystal forms of DEL-III (both light orange) superposed on HEL (PDB entry 1iee, grey). Amino-acid identities different from the chicken enzyme are shown as sticks. The arrow indicates the active-site cleft. (*c*–*h*) Ensemble superposed with the HEL–HyHEL5 complex (PDB entry 1yqv, cyan cartoon and surface) and the HEL–HyHEL10 complex (PDB entry 3d9a, slate cartoon and surface). (*e*) Position 68 is directly adjacent to HyHEL5, whilst positions 93 and 100 are directly adjacent to HyHEL10. (*f*) Substitution of Lys49 of the HyHEL10 light chain by threonine enhances binding fivefold to DELs which contain N97R. (*g*) The salt bridge found in the HEL–HyHEL5 complex is not supported by the R68K substitution in DELs. (*h*) The S100R substitution found in DEL-III disrupts binding to HyHEL10 owing to a steric clash with the HyHEL10 surface (shaded).

When superposed against a high-resolution HEL crystal structure (PDB entry 1iee; Sauter *et al.*, 2001 ▸), Pekin DEL-I and DEL-III, unsurprisingly, display the same C-type lysozyme fold (Figs. 7 ▸ *a* and 7 ▸ *b*). Amino-acid positions whose identities differ between chicken and duck isoforms map predominantly to surface areas distal to the active-site cleft (indicated by sticks and the arrow, respectively, in Figs. 7 ▸ *a* and 7 ▸ *b*).

The availability of DEL structural information now complements the rationalization of epitope-mapping data that was previously reported for certain antibodies with respect to the lysozymes purified from various species. For instance, the combination of sequence and binding discrepancies noted between the lysozymes purified from various Galliformes (chickens, ducks, quails and other heavy ground birds) enabled the lysozyme surface epitope for the classic HEL-binding antibody HyHEL5 to be predicted (Smith-Gill *et al.*, 1982 ▸). Specifically, it was noted that lysozyme position 68 was crucial for tight HyHEL5 binding; substitution from arginine to lysine, as found in duck lysozymes, was noted to weaken this interaction. The crystal structure of the complex formed between HEL and HyHEL5 has been solved and multiple versions of the complex have been refined (Sheriff *et al.*, 1987 ▸; Cohen *et al.*, 1996 ▸, 2005 ▸), revealing a crucial salt bridge between the guanidinium moiety of a fully extended Arg68 of HEL and Glu50 of the heavy chain of HyHEL5. Superposition of this complex onto our DEL ensemble (Figs. 7 ▸ *c*, 7 ▸ *d* and 7 ▸ *e*) reveals that even an extended lysine residue substituted at this position (as found in the duck structures) is incapable of forming the analogous salt bridge (Fig. 7 ▸ *g*). Two additional HyHEL5–lysozyme crystal structure complexes (both refined to ∼2.6 Å resolution) complement our duck-only lysozyme structures in this regard: that between HyHEL5 and the R68K mutant of HEL (PDB entry 2iff; Chacko *et al.*, 1995 ▸), and that between HyHEL5 and bobwhite quail egg lysozyme (BWQL), which only differs from HEL at four positions (compared with ∼20 in the case of DEL; see Fig. 6 ▸ for comparative homology percentages) but naturally contains the R68K mutation (PDB entry 1bql; Chacko *et al.*, 1996 ▸). As with the duck lysozyme ensemble, the other BWQL positional differences relative to HEL appear not to be involved in binding and, like the HEL R68K mutant, superpose equivalently with the duck lysozyme molecules with respect to the wild-type complex. Interestingly, both of these PDB entries model a bridging water molecule that connects R68K to Asp50 of HyHEL5, although the positioning (non-identical positions) and occupancy (if not validity) of such modelling is questionable given that disorder of Lys68 is noted in one structure (R68K-HEL) and both models appear to be overfitted [*R* and *R*_free_ of 0.19 and 0.29, respectively, for one and *R* of 0.18 (and no *R*_free_ reported) for the other], whilst two HyHEL5–HEL complex structures from the same group (and same vintage) have been superseded in the PDB by the lone 2005 entry (PDB entry 1yqv). In any case, the extent to which analogous bridging solvent would contribute to binding in the case of DEL-I and DEL-III can only be properly addressed by solving the co-crystal structures of the HyHEL5–duck lysozyme isoforms. It is also reported that affinity between HyHEL5 and R68K-HEL and BWQL decreases from a *K*_d_ of ∼1 × 10^−11^ *M* (for the wild-type HEL interaction) to ∼1 × 10^−7^ *M* in both cases (*i.e.* an ∼1000-fold decrease), as determined using a combination of PEG immunoprecipitation and ELISA methods (Padlan *et al.*, 1989 ▸). We also observed a significant decrease in affinity comparing the binding of HyHEL5 to HEL and to the duck lysozyme isoforms (∼50–100-fold decrease; Table 4 ▸). Although the duck lysozymes are distinct at the amino-acid level from those of R68K-HEL and BWQL (both with approximately 20 amino-acid differences), we suggest that the differences in the scales of the decreases in affinity are more likely to be a reflection of the sensitivity of the techniques employed rather than a reflection of gross differences in binding.

In addition to HyHEL5, the other anti-lysozyme antibody for which duck lysozyme binding data has been reported is HyHEL10, which binds to a different non-overlapping surface of lysozyme compared with HyHEL5 (Figs. 7 ▸ *c* and 7 ▸ *d*). Mutational analysis of the HyHEL10 antibody revealed that substitution of antibody light-chain position 49 from lysine to threonine increased the affinity of this antibody for duck lysozyme by fivefold (Lavoie *et al.*, 1992 ▸). Inspection of the superposed ensemble reveals that one of the ∼20 amino-acid differences between the chicken and duck enzymes is at position 93, where an asparagine on the surface of the chicken enzyme is replaced by an arginine residue (Fig. 7 ▸ *f*). Hence, the reason for the increased affinity noted for the K49T-substituted antibody is likely to be twofold: elimination of like positive charges that would otherwise be in close proximity and the provision of additional space on the surface of the antibody to accommodate the N93R substitution found on the surface of the duck enzymes. Additionally, as indicated by our binding data, one of the duck isoforms, DEL-III, failed to bind HyHEL10 with any substantial affinity (Table 4 ▸). Inspection of the structural superposition reveals that the additional arginine residue found in DEL-III (S100R) would be expected to sterically disrupt the intimate surface complementarity with HyHEL10 required for high-affinity binding (Fig. 7 ▸ *h*; the arginine side chain projects through the antibody surface), consistent with the noted elimination of binding. This observation exemplifies how even single mutations within the epitope of an antigen can enable evasion from a neutralizing antibody.

Apart from the complex of HyHEL10 with HEL, the only other HyHEL10-lysozyme complex for which a structure is available involves a mutant of HyHEL10 bound to lysozyme obtained from turkey egg (PDB entry 1uac; Kumagai *et al.*, 2003 ▸). Turkey egg lysozyme (TEL) has seven amino-acid differences with respect to HEL and ∼20 differences with respect to DEL (Fig. 6 ▸). Phage display using a restricted library of antibody fragments [limited to four positions within heavy-chain complementarity-determining region 2 (CDR2)] was used to select a HyHEL10 variant with three amino-acid substitutions in the heavy-chain CDR2 loop (T53S, S54F and Y58F) that resulted in a modest fivefold improvement in binding TEL relative to HEL (Nishimiya *et al.*, 2000 ▸; Kumagai *et al.*, 2003 ▸). Curiously, structural analysis revealed very little difference between the HyHEL10 triple mutant bound to either HEL or TEL, or in fact between these structures and the unmutated HyHEL10–HEL complex, leading the authors to posit that affinity is not simply a direct measure of the shape complementarity between the antigen and the antibody, which appeared to be roughly equivalent in all three cases. The only difference between TEL and DEL-I adjacent to the mutated surface of HyHEL10 is lysozyme position 101 (glycine in TEL, aspartate in DEL), although we predict that the aspartate side chain in DEL-I would not sterically preclude the binding of DEL-I to this HyHEL10 mutant. Additionally, as with HEL, TEL lacks the S100R substitution found in DEL-III, which acts as a steric impediment to HyHEL10 binding altogether (Fig. 7 ▸ *h*).

In conclusion, we have solved the crystal structures of C-type lysozymes DEL-I and DEL-III from Pekin duck. Verification of amino-acid sequences, including that of DEL-II, has been enabled by a combination of side chains being clearly resolved in electron density, prior amino-acid and DNA sequences, and peptide mass fingerprinting and whole-protein mass spectrometry. The structures/sequences corroborate prior findings regarding the affinity of duck enzymes for well characterized anti-lysozyme antibodies, thereby providing structural insights into commonly used immunological model systems.

## Supplementary Material

PDB reference: Pekin duck egg lysozyme isoform I, 5v8g

PDB reference: Pekin duck egg lysozyme isoform III, orthorhombic form, 5v92

PDB reference: Pekin duck egg lysozyme isoform III, cubic form, 5v94

Supplementary Figure S1.. DOI: 10.1107/S2059798317013730/cb5096sup1.pdf

## Figures and Tables

**Table 1:** Data collection and processing Values in parentheses are for the outer shell.

Crystal	DEL-I	DEL-IIIc	DEL-IIIo
Diffraction source	MX2, Australian Synchrotron	MX2, Australian Synchrotron	MX2, Australian Synchrotron
Wavelength (Å)	0.9537	0.9537	0.9537
Space group	*P*2_1_	*P*2_1_3	*P*2_1_2_1_2_1_
*a*, *b*, *c* (Å)	28.2, 65.4, 31.6	96.0, 96.0, 96.0	43.9, 58.2, 107.3
α, β, γ (°)	90.0, 113.2, 90.0	90.0, 90.0, 90.0	90.0, 90.0, 90.0
Resolution range (Å)	32.7–1.20 (1.22–1.20)	42.9–1.65 (1.68–1.65)	35.8–1.11 (1.13–1.11)
Total No. of reflections	143153 (6285)	741733 (33403)	859068 (37380)
No. of unique reflections	30531 (1459)	35458 (1804)	108789 (5355)
Completeness (%)	93.5 (88.6)	99.9 (100.0)	99.7 (100.0)
Multiplicity	4.7 (4.3)	20.9 (18.5)	7.9 (7.0)
〈*I*/σ(*I*)〉	10.2 (2.8)	18.6 (2.7)	13.3 (2.1)
*R*_r.i.m._	0.090 (0.547)	0.103 (1.043)	0.078 (0.882)
Overall *B* factor from Wilson plot (Å^2^)	9.9	19.4	8.6

**Table 2:** Structure solution and refinement

Crystal	DEL-I	DEL-IIIc	DEL-IIIo
Resolution range (Å)	32.7–1.20	30.34–1.65	29.08–1.11
No of reflections, working set	28891	33669	103168
No of reflections, test set	1613	1747	5531
Final *R*_cryst_	0.145	0.187	0.137
Final *R*_free_	0.180	0.215	0.162
No. of non-H atoms			
Total	1105	2182	2433
Protein	986	1978	2052
Ion/ligand	4	51	33
Water	115	153	348
R.m.s. deviations			
Bonds (Å)	0.0127	0.015	0.0105
Angles (°)	1.58	1.63	1.52
Average *B* factors (Å^2^)			
Protein	15.0	26.9	13.3
Ion/ligand	23.3	38.8	23.6
Water	29.1	32.9	26.9
Ramachandran plot			
Most favoured (%)	99.2	98.1	99.2
Allowed (%)	100.0	100.0	100.0
PDB code	5v8g	5v94	5v92

**Table 3:** Whole-protein mass spectrometry: predicted *versus* observed mono­isotopic masses in Da and their difference, together with the level of error in parts per million (p.p.m.) for HEL, DEL-I, DEL-II and DEL-III

Lysozyme	Predicted	Observed	Predicted − observed	Error (p.p.m.)
HEL	14295.82	14295.81	0.01	0.5
DEL-I	14391.90	14391.88	0.02	1.3
DEL-II	14460.96	14460.98	−0.02	−1.5
DEL-III	14589.08	14589.07	0.00	0.2

**Table 4:** Binding kinetics for the binding of duck and chicken lysozymes to immobilized HyHEL5 and HyHEL10 Fab arms showing the association rate constant (*k*_a_), the dissociation rate constant (*k*_d_) and the equilibrium dissociation constant (*K*_d_) Italicized data indicate parameters that are poorly determined (instrumentation limits), although it can be safely concluded that HyHEL10 binds HEL with high affinity and DEL-III poorly.

	HyHEL5			HyHEL10		
	*k*_a_ (s^−1^ *M* ^−1^)	*k*_d_ (s^−1^)	*K*_d_ (*M*)	*k*_a_ (s^−1^ *M* ^−1^)	*k*_d_ (s^−1^)	*K*_d_ (*M*)
HEL	2.2 × 10^5^	1.2 × 10^−4^	5.4 × 10^−10^	5.3 × 10^5^	*<1 × 10^−7^*	*≪1 × 10^−10^*
DEL-I	2.0 × 10^5^	8.1 × 10^−3^	4.1 × 10^−8^	1.6 × 10^5^	6.3 × 10^−3^	4.0 × 10^−8^
DEL-II	1.7 × 10^5^	7.0 × 10^−3^	4.1 × 10^−8^	1.7 × 10^5^	5.6 × 10^−3^	3.4 × 10^−8^
DEL-III	1.3 × 10^5^	3.1 × 10^−3^	2.5 × 10^−8^	1.7 × 10^4^	*∼6 × 10^−1^*	*≫1 × 10^−6^*
