# Supplementary material for: Structural basis of antigen recognition: crystal structure of duck egg lysozyme
Source: Acta Crystallogr D Struct Biol. 2017 Oct 25;73(Pt 11):910–20. doi: 10.1107/S2059798317013730 (PMC5683014; doi:10.1107/S2059798317013730)
Supplement: Supplementary file 1 [file d-73-00910-sup1.pdf]

STRUCTURAL BIOLOGY

Volume 73 (2017)

Supporting information for article:

Structural basis of antigen recognition: crystal structure of duck egg lysozyme

David Brent Langley, Ben Crossett, Peter Schofield, Jenny Jackson, Mahdi Zeraati, David Maltby, Mary Christie, Deborah Burnett, Robert Brink, Christopher Goodnow and Daniel Christ

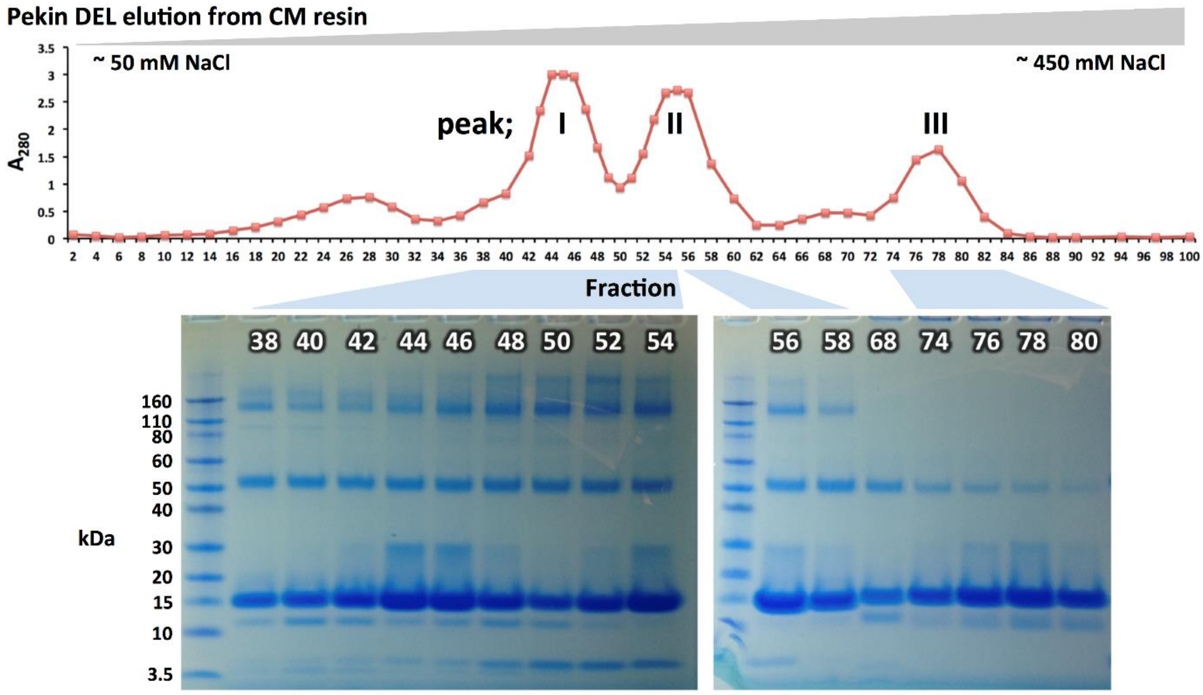

Figure S1 Elution profile of Pekin duck lysozymes from ion-exchange resin complemented by un-cropped SDS-PAGE examination of fractions.
